# Supplementary material for: Survival Pathways of HIF-Deficient Tumour Cells: TCA Inhibition, Peroxisomal Fatty Acid Oxidation Activation and an AMPK-PGC-1α Hypoxia Sensor
Source: Cells. 2022 Nov 14;11(22):3595. doi: 10.3390/cells11223595 (PMC9688062; doi:10.3390/cells11223595)
Supplement: Supplementary file 1 [file cells-11-03595-s001.zip › cells-2001626-supplementary.pdf]

SUPPLEMENTARY INFORMATION

METHODS

Measurement of acetate

Cells were briefly rinsed twice with ice-cold phosphate-buffered saline. 1 ml of ice cold 6% perchloric acid solution was added per plate and incubated for 5 minutes on ice. The cells were then scraped from the plate, and centrifuged at 1000rpm for 10 minutes at 4°C. The pH of the supernatant was adjusted to around 7 and the samples were centrifuged again, as previously described. The supernatant was then frozen in liquid nitrogen and freeze-dried. The lyophilizate was reconstituted in 0.6ml D_2O, and 0.5ml was transferred to a 5 mm NMR tube. For the ^{1}H MRS study, 25µl of 10mM sodium 3-trimethylsilyl-2,2,3,3-tetradeuterpropionate (TSP) was added to the samples and the pH was readjusted to 7. TSP served as an indicator both for chemical shift calibration and for quantification of metabolites. All acid and base solutions used for pH adjustment at this stage of the assay were diluted in D_2O rather than H_2O. ^{1}H MR spectra were obtained using a Bruker 600 MHz spectrometer.

^{13}C tracer studies

Cells were grown for 48 hours in pyruvate-free medium containing a 18mM [1,2-$^{13}C_2$]-D-glucose (>99% purity and 99% isotope enrichment for each carbon position; Sigma-Aldrich-Isotec) (n=4). The medium was then collected, cells were washed twice in phosphate-buffered saline, and cell pellets were harvested.

Metabolite extractions and analyses were performed as described previously.[31] Lactate was extracted from 100µL cell culture medium by ethylene chloride after acidification with HCl, derivatized to its propylamine-heptafluorobutyrate ester, and analysed by GC-MS. The *m/z* 328 (carbons 1–3 of lactate; chemical ionization) were monitored for positional mass isotopomer determination. Fatty acids were extracted by saponification of TRIzol cell extracts after removal of the RNA-containing supernatant with 30% KOH and 100% ethanol using petroleum ether. Fatty acids were then methylated using 0.5N methanolic-HCl. Myristate, palmitate and long chain saturated and mono-unsaturated oleate were measured using selected ion monitoring. The enrichment of acetyl units and the synthesis of new lipid fractions were determined using the mass isotopomers of palmitate, and the enrichment of ^{13}C-labelled acetyl units, as determined by mass

isotopomer distribution analysis, was used to assess synthesis, elongation, and desaturation of the new lipid fraction. Medium C^{13}:C^{12} ratios were measured in the released CO_2 and used to measure glucose and glutamine oxidation. RNA ribose was isolated by acid hydrolysis of cellular RNA after TRIzol purification of cell extracts, and derivatized to its aldonitrile acetate form using hydroxylamine in pyridine with acetic anhydride (Supelco) before mass spectral analyses. The ion clusters were monitored around the *m/z* 217 (carbons 3–5 of ribose) and *m/z* 242 (carbons 1–4 of ribose; electron impact ionization) to determine concentration and positional distribution of ^{13}C in ribose. Glycogen glucose was co-extracted and derivatized with RNA ribose by TRIzol but monitored at the ion clusters around the *m/z* 187 (carbons 3–6 of glycose) and *m/z* 242 (carbons 1–4 of glucose; electron impact ionization) to determine concentration and positional distribution of ^{13}C in glycogen glucose.

Glutamine was extracted from media as previously described[32] and the trifluoroacetyl butyl ester (TAB) of the glutamine and glutamate fraction was analysed using GC-MS. Under electron impact ionization conditions, ionization of TAB-glutamine/glutamate produces two fragments, *m/z198* and *m/z152,* corresponding to C2-C5 and C2-C4 of the carbon skeleton. Glutamine and glutamate labelled on the 4-5 carbon positions indicate pyruvate dehydrogenase activity, while labelling on the 2-3 carbon positions indicates entry of glucose carbons into the TCA via pyruvate carboxylase. TCA anabolic glucose utilization was calculated from the m_1/m_2 ratios of glutamate.[33] ^{13}C labelling patterns are shown in Figure S1a-c.

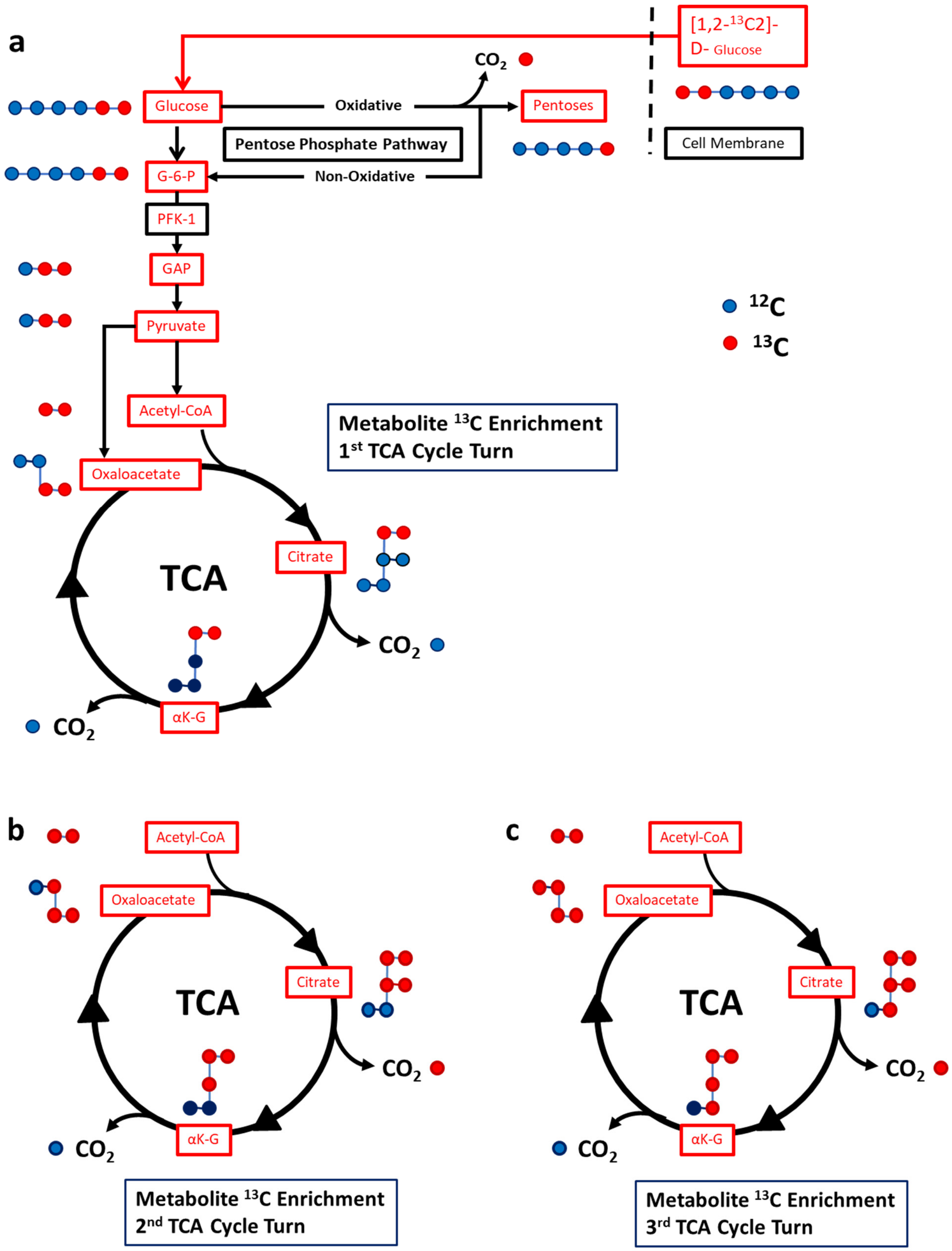

Figure S1. ^{13}C Enrichment patterns. a ^{13}C enrichment patterns after the first turn through the TCA cycle. **b** ^{13}C enrichment patterns after the second turn through the TCA cycle. **c** ^{13}C enrichment patterns after the third turn through the TCA cycle.
